# Development and validation of a nomogram for predicting significant coronary artery stenosis in suspected non-ST-segment elevation acute coronary artery syndrome with low-to-intermediate risk stratification

**DOI:** 10.3389/fcvm.2022.1013563

**Published:** 2022-12-19

**Authors:** Meixiang Chen, Pengfei Li, Yuekang Huang, Shuang Li, Zheng Ruan, Changyu Qin, Jianyu Huang, Ruixin Wang, Zhongqiu Lin, Peng Liu, Lin Xu

**Affiliations:** ^1^The First School of Clinical Medicine, Southern Medical University, Guangzhou, Guangdong, China; ^2^General Hospital of the Southern Theatre Command, Chinese People’s Liberation Army (PLA), Guangzhou, Guangdong, China; ^3^Zhujiang Hospital, The Second School of Clinical Medicine, Southern Medical University, Guangzhou, Guangdong, China; ^4^Branch of National Clinical Research Center for Geriatric Diseases, Chinese PLA General Hospital, Guangzhou, Guangdong, China

**Keywords:** prediction model, nomogram, non-ST-segment elevation acute coronary artery syndrome, low-to-intermediate risk, validation nomogram for predicting significant coronary artery stenosis

## Abstract

**Background:**

Patients with non-ST-segment coronary artery syndrome (NSTE-ACS) have significant heterogeneity in their coronary arteries. A better assessment of significant coronary artery stenosis (SCAS) in low-to-intermediate risk NSTE-ACS patients would help identify who might benefit from invasive coronary angiography (ICA). Our study aimed to develop a multivariable-based model for pretesting SCAS in suspected NSTE-ACS with low-to-intermediate risk.

**Methods:**

This prediction nomogram was constructed retrospectively in 469 suspected NSTE-ACS patients with low-to-intermediate risk. Patients were divided into a development group (*n* = 331, patients admitted to hospital before 1 May 2021) and a temporal validation group (*n* = 138, patients admitted to hospital since 1 May 2021). The outcome was existing SCAS, including left main artery stenosis ≥50% or any subepicardial coronary artery stenosis ≥70%, all confirmed by invasive coronary angiography. Pretest predictors were selected using Least Absolute Shrinkage and Selection Operator (LASSO) and stepwise logistic regression.

**Results:**

Derivation analyses from the development group (*n* = 331, admitted before 1 May 2021) generated the 7 strongest predictors out of 25 candidate variables comprising smoker, diabetes, heart rate, cardiac troponin T, N-terminal pro-B-type natriuretic peptide, high-density lipoprotein cholesterol, and left atrial diameter. This nomogram model showed excellent discrimination ability with an area under the receiver operating characteristic curve (AUC) of 0.83 in the development set and 0.79 in the validation dataset. Good calibration was generally displayed, although it slightly overestimated patients’ SCAS risk in the validation group. Decision curve analysis demonstrated the clinical benefit of this model, indicating its value in clinical practice. Furthermore, an optimal cut-off of prediction probability was assigned as 0.61 according to the Youden index.

**Conclusion:**

A prediction nomogram consisting of seven readily available clinical parameters was established to pretest the probability of SCAS in suspected NSTE-ACS patients with low-to-intermediate risk, which may serve as a cost-effective risk stratification tool and thus assist in initial decision making.

## Introduction

Patients with non-ST-segment coronary artery syndrome (NSTE-ACS) have significant heterogeneity in their coronary arteries, ranging from structurally normal vessels, varying degrees of atherosclerosis to extensive obstructive coronary artery disease (CAD), and invasive coronary angiography (ICA) is the gold standard for evaluation (1, 2). Current guidelines recommend an immediate invasive strategy (<2 h) for very high-risk patients and an early strategy (<24 h) for high-risk patients. For low-to-intermediate risk patients, either an elective ICA or a non-invasive ischemia-driven test can be performed (3, 4). Assessing the condition of the coronary arteries in patients with low-to-intermediate risk remains challenging. Recent studies have shown that approximately 20–30% of low-risk NSTE-ACS patients who underwent ICA had obstructive coronary stenosis (2, 5, 6). Although ICA is recognized as the most precise pathway for coronary pathology, given the complications and cost, non-invasive methods may be a more appropriate initial screening step for patients with low-to-intermediate risk stratification (5, 7).

There are several non-invasive imaging strategies for selecting patients with a high probability of coronary lesions (8, 9). However, the choice of the optimal test varies across individuals. Since multiple factors, such as accessibility, security, expertise, and cost, are needed for judgment and little evidence on comparative effectiveness has been reported (10), the decision to refer for ICA is sometimes based on factors other than non-invasive imaging. This could be influenced by clinicians’ subjective judgment and patient preference, resulting in inappropriate further treatment decisions (7). A straightforward, low-cost, and quantitative technique for identifying suitable ICA candidates is required (10), which should begin with precise risk prediction in patients with significant coronary artery stenosis (SCAS). Some well-established clinical multivariate risk models exist to pretest the likelihoods of obstructive CAD, such as the updated Diamond-Forrester risk score and Duke risk model (11–13). However, these predictive models were shown to overestimate the prevalence of obstructive CAD in the current population (14). Furthermore, there is no specific algorithm for detecting SCAS in NSTE-ACS patients with low-to-intermediate risk stratification. Based on these considerations, we aimed to generate a dedicated model for individualized SCAS prediction in such patients using readily available clinical parameters, which may add incremental value in the identification of patients with potential high-risk coronary artery stenosis and help to inform further decision-making.

## Materials and methods

### Study design

This research was an observational, cross-sectional study presented following the Transparent Reporting of a Multivariable Prediction Model for Individual Prognosis or Diagnosis (TRIPOD) statement. The study was approved by the Ethics Committee of Zhujiang Hospital of Southern Medical University, and written informed consent was obtained from each patient before ICA.

### Patients selection

**FIGURE 1:**
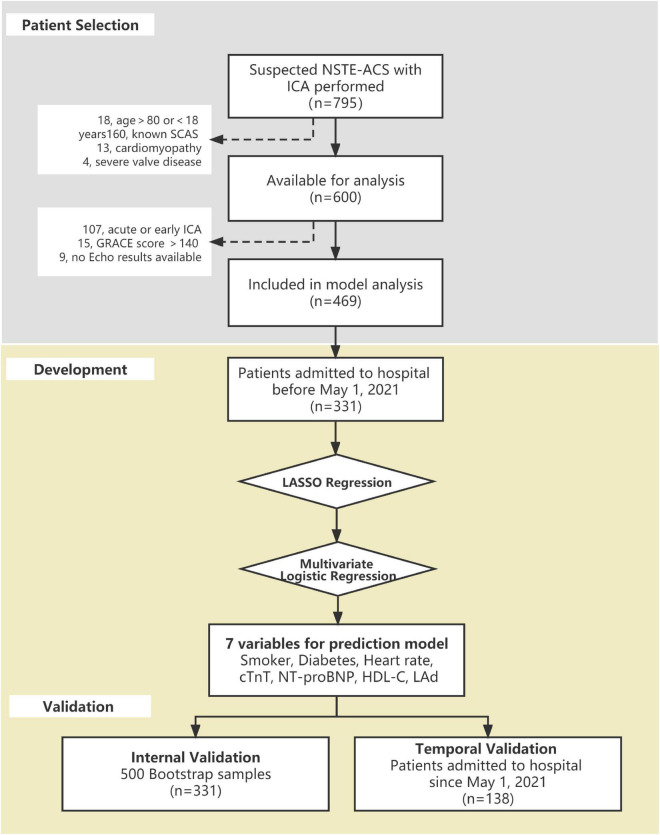
Flowchart of the process of development and validation of the significant coronary artery stenosis (SCAS) prediction nomogram.

In the study, 795 suspected NSTE-ACS patients with ICA performed during hospitalization between January 2019 and January 2022 were initially reviewed at Zhujiang Hospital of Southern Medical University, Guangdong, China. Additional inclusion criteria were low-to-intermediate risk patients who underwent ICA for the first time during that admission. The exclusion criteria were as follows: (1) age < 18 or > 80 years old; (2) confirmed diagnosis of CAD with or without previous revascularization by ICA; (3) assessed as high-risk or very high-risk (met the risk category listed in the 2020 ESC guidelines, details in the Supplementary material); (4) severe valve regurgitation or stenosis; (5) known congenital cardiac disease or cardiomyopathy; and (6) no echocardiography parameters available. Finally, a total of 469 patients were screened for prediction model analysis. Patients admitted to hospital before 1 May 2021 (*n* = 331) served as a development group, and 138 individuals admitted to hospital since that day were assigned for temporal validation. The process of population selection and study design is illustrated in Figure 1.

### Study outcome

The study outcome was defined as the presence of SCAS, including left main artery stenosis ≥50% or any subepicardial coronary artery stenosis ≥70%. Coronary slow blood flow, myocardial bridge, and collateral circulation were also recorded.

### Clinical data collection

We extensively collected the patients’ clinical information, including demographic characteristics, medical history, symptoms, vital signs on admission, biochemical markers, medications, the first electrocardiogram after admission, and the echocardiography parameters obtained before ICA. Trained researchers collected all the clinical information from standardized electronic case records, and every patient’s Global Registry of Acute Coronary Event (GRACE) score was calculated retrospectively. Medical history of hypertension or diabetes mellitus was defined as known or treatment with related medications. A smoker was described as having a documented history or current smoking. The electrocardiogram manifestations were mainly divided into the following categories: ST-segment depression, T-wave inversion, different types of arrhythmia, and normal or inconclusive changes. The size of the left atrial transverse meridian was measured on the standard four-chamber view. For some parameters tested repeatedly during hospitalization, such as cardiac troponin T (cTnT), the results obtained on admission were collected for analysis. Two cardiologists independently interpreted all angiographic images, blinded to other clinical information. Cases with inconsistent results were judged by another senior expert.

### Model development

For model development, 25 candidate predictors were preliminarily selected based on clinical experience, related literatures, easy accessibility, and the degree of missing data. Ten general risk factors, including sex, age, smoking, diabetes, hypertension, previous stroke, alcohol use, heart rate, systolic blood pressure, and diastolic blood pressure on admission, were included. Twelve biomarkers and three indicators obtained from electrocardiogram and echocardiography were considered; all the items are listed in Table 1. Given the dimensional differences between continuous variables and for the simple-to-use sake, we classified several continuous parameters into several interval ranges and run them as dumb variables in the analysis. We grouped heart rate, cTnT, and N-terminal pro-B-type natriuretic peptide (NT-proBNP) on the basis of the clinical reference value intercept points of each variable. We categorized high-density lipoprotein cholesterol (HDL-C) and left atrial diameter (LAd) according to quartile range and 90th percentile, respectively. Due to a large number of candidate variables and small samples, a penalty function-based regression technique, Least Absolute Shrinkage and Selection Operator (LASSO), was applied to preliminarily screen out the valuable predictors. Then, those variables were further fitted *via* stepwise backwards selection based on Akaike’s information criterion to generate the most parsimonious prediction model. We used the XGBoost model and Wald value to calculate the relative importance of the final variables.

**TABLE 1:** Characteristics of the population for derivation and validation sets.

Characteristics	Derivation set (*n* = 331)	Validation set (*n* = 138)	*t*/χ^2^/*Z* value	*P-*value
**Basic condition and medical history**				
Male	234 (70.7%)	97 (70.3%)	0.008	1.000
Age, mean (SD), y	60.38 (10.64)	59.81 (10.87)	0.527	0.598
Heart rate, mean (SD), b.p.m.	78.08 (12.33)	79.49 (10.97)	–1.157	0.248
Systolic blood pressure, mean (SD), mm Hg	135.50 (19.57)	137.80 (16.96)	–1.726	0.085
Diastolic blood pressure, mean (SD), mm Hg	77.91 (12.09)	77.11 (13.46)	0.634	0.526
Smoker	149 (45.0%)	19 (13.8%)	41.364	<0.001
Hypertension	184 (55.6%)	74 (53.6%)	0.152	0.760
Diabetes	73 (22.1%)	28 (20.3%)	0.179	0.672
Previous stroke	24 (7.3%)	10 (7.2%)	0.000	1.000
**ECG**				
Normal or inconclusive	132 (39.9%)	72 (52.2%)	14.364	0.001
TWI or AR	101 (30.5%)	47 (34.0%)		
STD	98 (29.6%)	19 (13.8%)		
**Lab results**				
**cTnT, μg/L**				
<0.014	205 (61.9%)	103 (74.6%)	7.135	0.028
≥0.014, <0.028	31 (9.4%)	7 (5.1%)		
≥0.028	95 (28.7%)	28 (20.3%)		
**NT-proBNP, ng/dL**				
<300	264 (79.8%)	113 (81.9%)	3.551	0.312
≥300, <600	23 (6.9%)	14 (10.1%)		
≥600, <1,200	22 (6.6%)	6 (4.3%)		
≥1,200	22 (6.6%)	5 (3.6%)		
Creatine kinase, median (IQR), IU/L	100.50 (68.00, 164.00)	94.12 (66.00, 142.50)	–1.098	0.272
Creatine kinase MB isoform, median (IQR), IU/L	13.10 (10.80, 18.00)	15.46 (10.44, 17.53)	–0.041	0.967
HbA1c, median (IQR),%	5.90 (5.50, 6.40)	5.90 (5.48, 6.30)	–0.397	0.691
Glucose, median (IQR), mmol/L	5.82 (5.15, 5.82)	5.53 (4.91, 7.39)	–1.690	0.091
Total cholesterol, median (IQR), mmol/L	4.88 (4.02, 5.78)	4.74 (3.90, 5.52)	–1.156	0.248
Triglycerides, median (IQR), mmol/L	1.56 (1.05, 2.32)	1.55 (1.08, 2.47)	–0.261	0.794
HDL cholesterol, median (IQR), mmol/L	1.16 (0.95, 1.35)	1.08 (0.91, 1.28)	–1.635	0.102
LDL cholesterol, median (IQR), mmol/L	2.89 (2.18, 3.65)	2.75 (2.27, 3.59)	–0.694	0.488
Homocysteine, median (IQR), μmmol/L	11.73 (9.84, 14.42)	11.30 (9.40, 14.09)	–0.960	0.337
Serum uric acid, mean (SD), μmmol/L	384.66 (105.00)	373.77 (108.11)	1.015	0.311
ALAT, mean (SD), IU/L	23.38 (14.07)	22.12 (14.64)	1.580	0.115
ASAT, mean (SD), IU/L	25.71 (19.18)	21.71 (12.84)	1.554	0.121
eGFR, mean (SD), mL/min	83.62 (19.40)	85.56 (19.13)	–0.058	0.953
Creatinine, mean (SD), μmmol/L	81.28 (20.30)	78.07 (21.15)	1.539	0.125
Albumin, mean (SD), g/L	42.79 (6.05)	42.90 (4.22)	–0.235	0.814
**Medication**				
Nitrates	88 (26.6%)	17 (12.3%)	11.410	0.001
Platelet inhibitors	306 (92.4%)	115 (83.3%)	8.806	0.004
Statins	264 (79.8%)	110 (79.7%)	0.000	1.000
β-blockers	216 (65.3%)	65 (47.1%)	13.367	<0.001
Anticoagulant	114 (34.4%)	19 (13.8%)	20.488	<0.001
Calcium channel blockers	99 (29.9%)	60 (43.5%)	8.002	0.005
ACEI or ARB	123 (37.2%)	48 (34.8%)	0.238	0.674
**Echocardiography**				
E/A, mean (SD)	1.04 (0.48)	0.99 (0.39)	0.969	0.333
LVEF, mean (SD),%	57.92 (6.18)	59.42 (4.25)	–2.614	0.009
IVST, mean (SD), mm	9.73 (1.36)	9.78 (1.63)	–0.323	0.747
LVPWT, mean (SD), mm	9.68 (1.35)	9.55 (1.53)	1.012	0.312
LVId, mean (SD), mm	43.96 (5.34)	43.74 (4.67)	0.421	0.674
RVId, mean (SD), mm	19.97 (3.28)	20.83 (2.69)	–2.716	0.007
LAd, mean (SD), mm	32.78 (5.20)	33.51 (4.60)	–1.438	0.151
RAd, mean (SD), mm	29.77 (4.20)	30.00 (3.92)	–2.134	0.033
WMSI, median (IQR)	1.00 (1.00, 1.12)	1.00 (1.00, 1.00)	–5.488	<0.001
GRACE Score, mean (SD)	104.06 (21.10)	97.93 (20.70)	2.884	0.004
	

SD, standard deviation; IQR, interquartile range; TWI, T wave inversion; AR, arrhythmia; STD, ST-segment depression; cTnT, cardiac troponin T; eGFR, estimated glomerular filtration rate; ALAT, alanine aminotransferase; ASAT, aspartate aminotransferase; ACEI, angiotensin-converting enzyme inhibitors; ARB, angiotensin receptor blockers; E/A, early diastolic peak velocity over late diastolic peak velocity of mitral orifice; IVST, interventricular septum thickness; LVPWT, left ventricular posterior wall thickness; LVId, left ventricular diastolic dimension; LAd, left atrial diameter; RAd, right atrial diameter; WMSI, left ventricular wall motion score index; GRACE, the Global Registry of Acute Coronary Event.

### Model validation and clinical utility

The model was internally validated using 500 bootstrap samples, and the temporal validation of the model was carried out in the later admission population (*n* = 138, approximately 30% of the total datasets). The predictive performance of the model was evaluated by discrimination and calibration in both the development and validation groups. Discrimination performances of the prediction model were reported using an area under the receiver operating curve (AUC) with a 95% confidence interval (CI). Calibration analyses were estimated by fitting the calibration curve between the nomogram predicted probability and actual observed condition, and we interpreted the model calibration curve through visual observation combined with clinical characteristics of the index. The clinical applicability of the nomogram was assessed using decision curve analysis (DCA), which presented the individual net benefits at different decision threshold probabilities. Through this curve, we were able to determine the most favorable probability interval for the model to predict SCAS. Furthermore, the Youden index was used to establish the best cut-off value.

### Statistical analysis

Statistical analyses for the model construction and nomogram production were performed in Empowerstats version 2.2 based on the R-work (R Project for Statistical Computing, Vienna, Austria). Baseline characteristic comparisons were carried out using SPSS^®^ Statistics 20 (IBM, Armonk, NY, USA). Variable distribution types were determined by the Kolmogorov–Smirnov test. Continuous measures are presented as the mean and standard deviation (SD) or median and interquartile range (IQR). An independent sample t test or the Mann–Whitney U test was used for comparisons between groups. Categorical features were expressed as frequencies with percentages and compared using Fisher’s exact tests. A *P*-value < 0.05 was considered statistically significant. Missing data were assumed to be randomly missing and completed using multiple imputations by chained equations. Missing values were imputed based on all candidate predictors and the outcome.

## Results

### Study population and characteristics

A total of 331 patients (60.38 ± 10.64 years, 70.7% male) and 138 patients (59.81 ± 10.87 years, 70.3% male) were enrolled in the derivation and validation datasets, respectively. Table 1 compares the baseline characteristics of the derivation group and the temporal validation group. The general condition of admission and medical history were similar between both sets. Patients in the validation group had lower GRACE scores, cTnT values, and wall motion score index than those in the development group but a greater left ventricular evaluation fraction. Defined ST-segment depression presented more frequently in the development group. Furthermore, there was a higher prevalence of cardiovascular-related drug (except for calcium channel blockers) use in the derivation set. In the development group, 52.3% of patients developed SCAS, compared to 38.4% in the validation group (*P*-value < 0.001). Supplementary Table 1 shows the baseline comparison of patients with SCAS and those without SCAS.

### Model development

**FIGURE 2:**
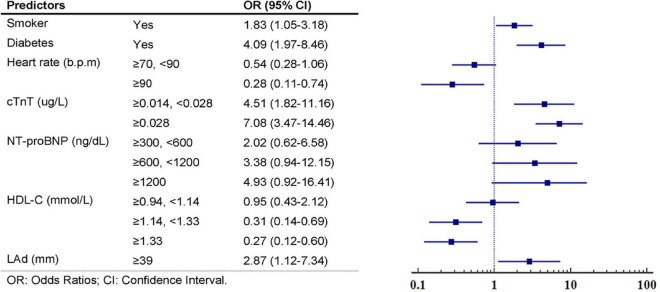
Multivariate logistic regression analyses and constructed multivariable predictors’ respective weights in the significant coronary artery stenosis (SCAS)-nomogram from the derivation set.

**FIGURE 3:**
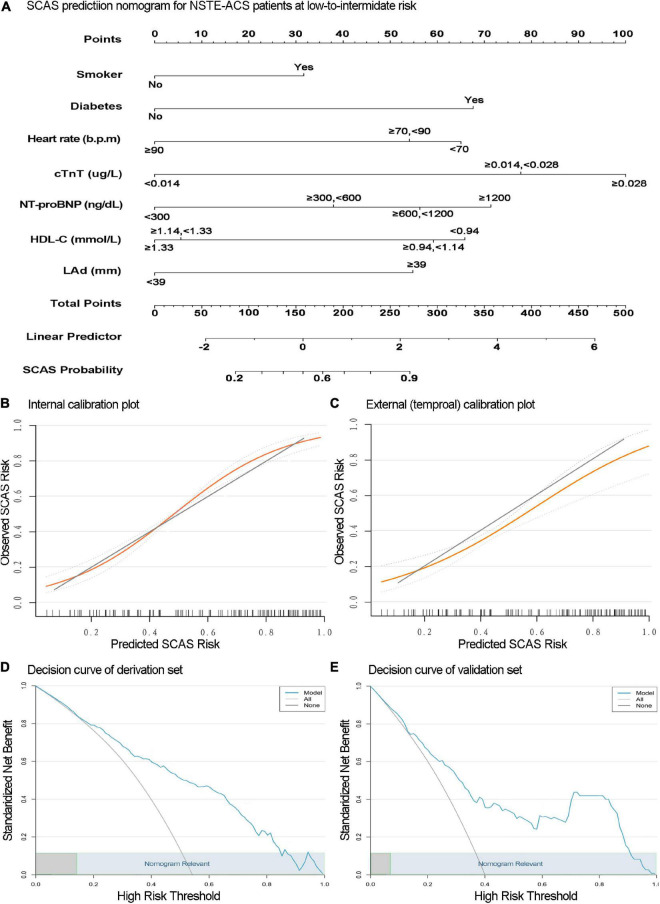
The nomogram for significant coronary artery stenosis (SCAS) prediction is illustrated **(A)**. Calibration plots depict the observed incidence of SCAS vs. the predicted probability of SCAS in the derivation **(B)** and validation **(C)** sets. The *x* and *y*-axes are the predicted probability and actual outcome, respectively. The green diagonal represents the prediction of the ideal model, the yellow line represents the prediction probability, and the light blue dotted line on both sides represents the 95% confidence interval of each predicted value. Decision curve analysis of the SCAS predictive nomogram in the derivation **(D)** and validation **(E)** sets. The *x*-axis is the threshold probability, and the *y*-axis represents the net benefit. The solid blue line depicts the decision-making benefits of the prediction model under different threshold probability values. The light gray negative slope curve assumes that all NSTE-ACS with low-to-intermediate risk will experience SCAS. The horizontal black line represents the assumption that no patients will experience SCAS. This nomogram showed (high) net benefit for wide thresholds (light blue rectangular area range).

After LASSO regression with the penalty coefficient “lambda” of 0.0224, 25 primary variables were reduced to 14 based on the derivation set. Supplementary Figure 1 contains a complete illustration of the procedures. The predictors were further screened using multivariate logistic regression. Seven predictors, namely, smoker, diabetes, heart rate, cTnT, NT-proBNP, HDL-C, and LAd, were kept in the final model due to the significance of the predictive value of variables (*P*-value < 0.1) and clinical relevance. The findings of the univariable and multivariate analyses of the LASSO-selected variables in the derivation set are summarized in Table 2. The seven predictors were then fitted into a multivariable model, with no one being deleted following stepwise backwards selection; details are shown in Figure 2 and Supplementary Table 2. Figure 3A graphically depicts each predictor’s effect on the risk of SCAS in the form of a nomograms.

**TABLE 2:** Univariable and multivariate analyses of the LASSO-selected variables in derivation set.

	Univariable model		Multivariable model		Included in the model
	OR(95% CI)	*P-*value	OR(95% CI)	*P-*value	
Age	1.02 (1.00, 1.05)	0.025	1.01 (0.98, 1.04)	0.489	
Diabetes	3.63 (2.02, 6.51)	<0.001	3.60 (1.53, 8.47)	0.003	Yes
Hypertension	1.79 (1.16, 2.78)	0.009	1.11 (0.61, 2.02)	0.732	
Smoker	2.12 (1.36, 3.30)	0.001	1.60 (0.88, 2.89)	0.092	Yes
Heart rate (b.p.m)					Yes
≥70, <90	0.86 (0.50, 1.48)	0.596	0.56 (0.28, 1.12)	0.102	
≥90	0.52 (0.25, 1.09)	0.083	0.25 (0.09, 0.68)	0.007	
ECG					
TWI or AR	1.46 (0.87, 2.46)	0.155	0.95 (0.50, 1.81)	0.875	
STD	3.73 (2.13, 6.52)	<0.001	1.06 (0.50, 2.26)	0.871	
cTnT (μg/L)					Yes
≥0.014, <0.028	5.43 (2.40, 12.29)	<0.001	3.44 (1.34, 8.80)	0.010	
≥0.028	9.78 (5.31, 18.02)	<0.001	7.06 (3.34, 14.93)	<0.001	
NT-proBNP (ng/dL)					Yes
≥300, <600	3.45 (1.32, 9.03)	0.012	1.87 (0.54, 6.51)	0.323	
≥600, <1,200	5.48 (1.81, 16.64)	0.003	3.41 (0.90, 12.95)	0.072	
≥1,200	7.72 (2.23, 26.71)	0.001	3.62 (0.67, 19.66)	0.137	
HDL-C (mmol/L)					Yes
≥0.94, <1.14	0.77 (0.39, 1.49)	0.435	1.00 (0.47, 2.13)	0.996	
≥1.14, <1.33	0.34 (0.17, 0.65)	0.001	2.81 (1.30, 6.08)	0.009	
≥1.33	0.28 (0.15, 0.53)	<0.001	2.91 (1.28, 6.62)	0.011	
Creatinine (μmmol/L)	1.02 (1.01, 1.03)	0.001	1.01 (0.99, 1.02)	0.284	
Total cholesterol (mmol/L)	0.88 (0.73, 1.04)	0.138	0.85 (0.67, 1.06)	0.153	
Homocysteine (μmmol/L)	1.12 (1.05, 1.19)	0.001	1.05 (0.97, 1.14)	0.228	
HbA1c (%)	1.58 (1.24, 2.01)	0.001	1.19 (0.87, 1.62)	0.276	
LAd (mm)					Yes
≥39	2.05 (0.96, 4.35)	0.062	2.58 (0.98, 6.81)	0.055	

OR, odds ratio; CI: confidence interval.

### Relative importance

**FIGURE 4:**
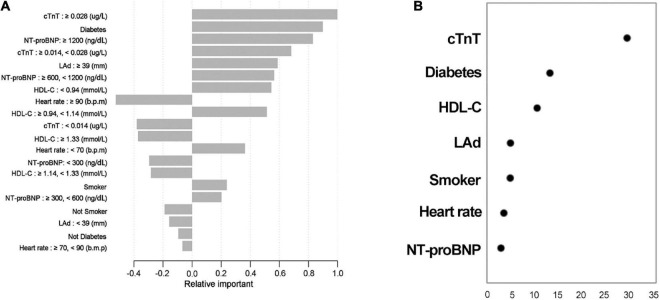
Relative contributions of each predictor. The results *via* XGBoots **(A)**; results obtained by calculating the Wald value **(B)**, which is measured as the square of (coefficients divided by the standard error). Variables are arranged in order of relative importance from top to bottom.

The proportional importance of each predictor in the machine learning model is shown in Figure 4A. The order of importance of the independent variables to distinguish whether there was a SCAS was as follows: cTnT, diabetes, NT-proBNP, LAd, HDL-C, heart rate, and smoker. In addition, the Wald value was determined to determine the relative contribution, which was derived as the square of the coefficients divided by the standard error. Figure 4B shows that cTnT and diabetes were the two variables with the most significant weight. Although the results of the two methods differed, they both revealed that cTnT and diabetes were the most important factor in the model equation.

### Model validation

**FIGURE 5:**
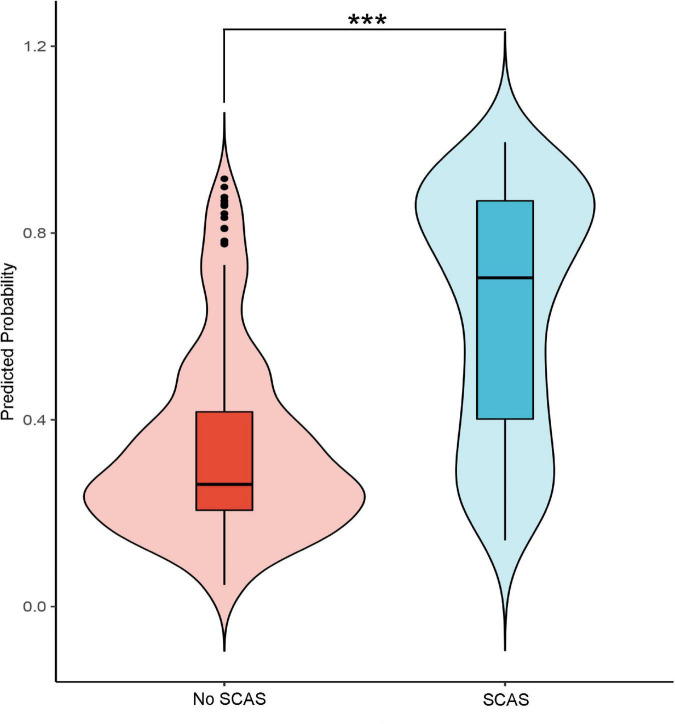
The probability distribution predicted by the model for patients in the group with significant coronary artery stenosis (SCAS) and patients in the non-SCAS group (***p-value < 0.01).

The internal validation with 500 bootstrap samples of the development set revealed a C-statistic of 0.83 (95% CI, 0.78–0.87). The calibration plot presented a good level of agreement between the observed outcomes and predicted results (Figure 3B). The SCAS-nomogram was temporally validated in 138 independent patients (admitted to the hospital after 1 May 2021) and yielded a C-statistic of 0.79 (95% CI, 0.72–0.86), indicating good discrimination ability. It can be seen from the prediction probability distribution plot that the model’s ability to distinguish between SCAS patients is relatively robust (Figure 5). Figure 3C shows that the prediction probability *via* the nomogram was mostly in accordance with the actual probability trend, but there was a modest tendency to overestimate the risk.

### Clinical utility

We assessed the clinical applicability of this SCAS-nomogram using decision curve analysis. As illustrated in Figures 3D,E, our prediction model displayed an excellent net benefit throughout practically the full range of threshold probabilities in both the derivation and validation sets, suggesting specific value in clinical practice regardless of any SCAS threshold. Furthermore, Table 3 depicts the performance of the nomogram at various cut-off values derived from the development set. Values of specificity, sensitivity, Youden index, positive predictive value, and negative predictive value were calculated. The best prediction probability threshold in the development set was 0.611, with a maximum Youden index of 0.528.

**TABLE 3:** Values for specificity, sensitivity, PPV, NPV, and Youden index of the predicted probability at different cut-off values.

Predicted probability^a^	Derivation set					Validation set				
	Specificity (%)	Sensitivity (%)	PPV (%)	NPV (%)	Youden index	Specificity (%)	Sensitivity (%)	PPV (%)	NPV (%)	Youden index
0.178	0.285	0.954	0.594	0.849	0.239	0.188	0.962	0.425	0.888	0.313
0.277	0.437	0.919	0.641	0.831	0.356	0.423	0.792	0.462	0.765	0.227
0.391	0.665	0.821	0.728	0.772	0.485	0.718	0.660	0.593	0.772	0.365
0.441	0.722	0.792	0.757	0.760	0.513	0.753	0.623	0.611	0.762	0.373
0.598	0.880	0.647	0.855	0.695	0.527	0.882	0.528	0.737	0.750	0.487
0.611^b^	0.886	0.642	0.860	0.693	0.528^c^	0.882	0.528	0.737	0.750	0.487
0.704	0.911	0.543	0.870	0.646	0.455	0.906	0.433	0.742	0.720	0.462
0.802	0.937	0.468	0.890	0.617	0.405	0.953	0.358	0.826	0.704	0.530
0.914	0.987	0.231	0.952	0.540	0.219	0.989	0.075	0.800	0.632	0.432
	

PPV, positive predictive value; NPV, negative predictive value.

^*a*^Based on the population of derivation set.

^*b*^The optimal cut-off value determined by Youden index.

^*c*^Maximum value of the Youden index.

### Model comparison to other risk scores

The SCAS-nomogram (AUC, 0.83) was a better prediction model than both updated Diamond-Forrester Model (UDFM; AUC, 0.60) and Duke risk score (DCS; AUC, 0.55). We calculated continuous Net reclassification improvement (NRI) for UDFM/SCAS-nomogram and DCS/SCAS-nomogram. Compared with UDFM or DCS, the NRI of SCAS-nomogram models both exceeded 20%. The results were similar to validation set-based analysis, details in Supplementary Table 3.

## Discussion

We developed and temporally validated a nomogram-illustrated model to generate individualized risk estimates specifically for SCAS in suspected NSTE-ACS patients with low-to-intermediate risk. Six basic clinical indicators and one simple two-dimensional echocardiographic parameter were included in the nomogram model. All of the variables are readily available or easily recorded by history consultation. The model can be used to assess the pretest probability of suspected NSTE-ACS in patients with a low-to-intermediate risk stratification who are considered for ICA or further non-invasive imaging procedures.

Some patients admitted to the hospital with suspected NSTE-ACS were considered low-to-intermediate risk. However, some of them actually present with SCAS needing further ICA or even revascularization (11). Identifying SCAS in low-to-intermediate risk patients is appealing, as it may reduce the potential incidence of acute myocardial infarction, refractory angina pectoris, and heart failure (15). Therefore, it is essential to identify those with SCAS and rule out who is in a good coronary state, which is pivotal for implementing preventive measures or deciding on a rapid hospital discharge. There are a variety of non-invasive imaging modalities to investigate the coronary artery condition in patients with suspected low-risk NSTE-ACS (9, 16). However, various items must be carefully considered when deciding which test to use, including available technology and expertise, relevant contraindications, patient preference, and potential complications or risks (7, 8). It is not clear how to best integrate these tests into patient care, and there is no suggested choice for the non-invasive evaluation of medium- and low-risk patients (9, 10). Hence, there is a need to provide a simple, low-cost, and quantitative assessment tool to quantify and differentiate individual SCAS risk profiles.

Clinical predictive models have been derived to estimate the likelihood of coronary artery stenosis. Pryor et al. (17) developed an 11-characteristics-based model named Duke risk score for predicting severe coronary state in symptomatic patients referred for suspected CAD. Then, they updated the model with chest X-rays to identify more complex coronary pathological anatomy types involving obstructive left main CAD, left anterior descending branch stenosis, and 3-vessel CAD (18). Similar to the Duke score, a five-variable-based predictive model reported by Hubbard et al. (19) as the coronary risk scale also generated a better-refined risk stratification. The most widely used CAD pre-test evaluation tool was Diamond-Forrester model, a simple but elegant model considering age, sex and type of chest pain. The models mentioned were of great value, however, they are somewhat outdated, as the epidemiological characteristics, treatment options, and diagnostic tools for cardiovascular disease have tremendously changed. Diamond-Forrester model has been updated using newly collected data and the range of age was also extended beyond 70 years old (20). In the recent past, James J et al.(11) published two models that were superior to traditional risk models for different degrees of coronary artery stenosis risk pretesting. However, unlike usual, coronary computed tomographic angiography was taken as a diagnostic reference rather than ICA in James’ study. More recently, some new indicators (such as coronary artery calcium score, gene expression score, and epicardial fat volume) have been proposed to improve CAD predictions (12, 21, 22), but they are not yet broadly applicable, as the key predictors are not routinely available. In addition, the existing coronary-related predictive model shared the limitation that they are not dedicated to identifying SCAS in suspected NSTE-ACS patients with low-to-intermediate risk.

Our SCAS-PREDICTION nomogram has several strengths. We quantified the probability of SCAS in low-to-intermediate risk NSTE-ACS patients without known CAD, and the outcome we observed takes into account the needs of ICA decision-making. Furthermore, robust variable selection techniques involving LASSO regression and standard multivariate analyses were adopted. A seven-element nomogram was built and validated internally and temporally, showed accurate individualized quantification of SCAS risk. Although, the prevalence of strongly weighted prognostic risk factors, such as smoking and level of cTnT was lower in the validation set compared with the derivation set. This resulted in a noticeable low risk of SCAS in the validation cohort, which brought some challenges to the generalization of the model. Still, this nomogram model shown decent discrimination in the both development and validation cohort with AUC of 0.83 and 0.79, respectively. In addition, our nomogram model was more predictive of SCAS than both UDFM and DCS, the optimal risk threshold was recommended as 0.61, which is much more convenient for application in practice.

This model included seven clinical predictors: smoker, diabetes, heart rate, cTnT, NT-proBNP, HDL-C, and LAd. Smoking and diabetes are two classic predictors presented in many risk prediction models for CAD purposes (11, 21). The potential mechanism of diabetes as a risk factor for cardiovascular disease may be due to the fact that hyperglycemia affects the coronary microvascular pathology, inflammation and sympathetic nervous system activity, vasospasm and circulatory structural remodeling, resulting in an increase in cardiovascular disease. HDL-C and NT-proBNP were valuable factors for SCAS prognosis, in line with previous findings (23–25). There is emerging evidence that high density-lipoprotein can reduce diabetes-related vascular complications through various pathways, low HDL-C is a residual and independent risk factor associated with coronary artery stenosis, myocardial infarction, increased risk of cardiovascular death who achieve optimal control of low-density lipoprotein cholesterol (26, 27). Despite the plain elevation of HDL-C level has not proved to be able to protect against cardiovascular disease, the clinical and epidemiological investigations have documented the inverse relationship between HDL-C and CAD. Cardiac troponin T was the most powerful predictor in our model, and its prognostic relevance in acute myocardial infarction has been widely demonstrated in many studies. Although cardiomyocyte necrosis is considered necessary for the release of cTn, many studies have suggested that reversible ischemia caused by coronary artery stenosis might play a role in the pathophysiology of myocardial injury and troponin elevation (28–30). Studies have proposed the mechanisms of cTnT elevation in NSTE-ACS, one caused by transient thrombotic coronary occlusion at the culprit lesion, and the other might be microcirculatory spasm caused by some particular blood components, such as serotonin, thromboxane, and endothelin (31). It is notable that admission heart rate was inversely related to the risk of SCAS, which may reflect the confounding effect of unmeasured heart rate-lowering medication or the limited sample size. Heart rate is one of the earliest and most readily available physiological parameters for outpatient and emergency patients and easy to interpretation or analysis.

A U-shaped relationship was found between in-hospital heart rate and mortality in patients with STEMI and NSTE-ACS in the results from 58 European hospital (32). While, there was no such U-shaped relationship in our data, which may be related to the different risk stratification level of the subjects and the small number of severe abnormal heart rate. In addition to the parameters mentioned above, an obviously enlarged left atrium (≥39 mm) was also included in the nomogram. The structural changes of the left atrial cavity have been previously linked to the slow flow of coronary arteries since insufficient oxygen supply leads to diastolic dysfunction of the left ventricle and elevates ventricular filling pressure, which subsequently conducted to the left atrium, causing increased atrial wall tension (33, 34). The metric LAd obtained from routinely available echocardiograms was simple and stable. Integrating this indicator is facile and brings more robust prognostic risk prediction.

Medical resources are becoming tenser while healthcare demands are rapidly increasing. To this effect, it is important to assess the relative effectiveness of diagnostic strategies not only in safety and accuracy but also in efficiency and cost. To our knowledge, no dedicated SCAS risk predicting model has been developed specifically for suspected NSTE-ACS patients without definite high risk. The most appealing aspect of our nomogram model is its specific population targeting and clinical ease of use. The findings could be utilized as incremental decision-making guidance, such as ICA for patients with a high probability of SCAS or on-demand follow-up for those in a good coronary artery state. Furthermore, it may help individuals raise awareness of their underlying disease and reap potential benefits from a primary prevention perspective.

## Study limitations

Although the development and validation datasets were distinguished independently by setting the cut-off point of admission time, the interpretability of temporal verification may be constrained since our study population was collected from a single unit. Furthermore, the full implementation of the coronary stent and balloon collection regulations in the area after February 2021 may cause some low-risk patients with NSTE-ACS to directly choose ICA due to the bonus of health insurance policy, resulting in a slight overestimation of SCAS risk in the validation group. Furthermore, after February 2021, when the coronary stent and balloon collection regulations are fully implemented in the area, some low-risk patients with NSTE-ACS may choose ICA directly due to a health insurance policy benefit, resulting in a slight overestimation of SCAS risk in the validation group. We acknowledge that future multicenter prospective validations are necessary to further test this model. It should be noted that this nomogram is only useful in cases where relevant predictor data are readily available. We balanced the clinical relevance and accessibility of the indicators as much as possible in the preliminary screening stage, and thus, the final predictive variables were easy to obtain. Although many baseline clinical variables were considered in the analysis, undoubtedly, we were unable to exclude the omission of other potentially more valuable variables that could optimize the risk prediction. To derive a simple and easy-to-use nomogram, we transformed some continuous variables into categorical variables, which might lessen the prediction ability of some parameters. Nonetheless, our model yielded good AUC in both the derivation and validation groups. Finally, more sophisticated models, such as those that use machine learning, may improve the model performance to some extent. On the other hand, regular variable selection approaches have the advantage of being straightforward to understand and adopt in practice.

## Conclusion

We developed the first SCAS risk prediction nomogram in suspected NSTE-ACS patients with low-to-intermediate risk, including seven routine clinical parameters that can be readily and quickly calculated and are easy to implement in practice. It can serve as a pretest algorithm for generating individualized estimates of the risk of SCAS and as an auxiliary tool to discriminate patients who might benefit from early discharge or further ICA. For individuals with a predicted chance of SCAS greater than 0.61, we recommend a further high specification non-invasive examination, even ICA.

## Data availability statement

The raw data supporting the conclusions of this article will be made available by the authors, without undue reservation.

## Ethics statement

The studies involving human participants were reviewed and approved by the Ethics Committee of Zhujiang Hospital, Southern Medical University. The patients/participants provided their written informed consent to participate in this study.

## Author contributions

MC and LX took responsibility for the integrity of the work as a whole, from inception to published article, and agreed to be accountable for all aspects of the work in ensuring that questions related to the accuracy or integrity of any part of the work are appropriately investigated and resolved. MC, PFL, PL, and LX performed conception and design. MC, PFL, PL, YH, SL, and RW performed acquisition, analysis, or interpretation of data. MC performed drafting of the work. ZR and CQ provided technical or material support. LX, PL, and ZL revised the manuscript critically for important intellectual content. All authors read and approved the final manuscript.

## References

[1] Linde J Kelbaek H Hansen T Sigvardsen P Torp-Pedersen C Bech J Coronary CT angiography in patients with non-ST-segment elevation acute coronary syndrome. *J Am Coll Cardiol.* (2020) 75:453–63. 32029126 10.1016/j.jacc.2019.12.012

[2] Kofoed K Kelbaek H Hansen P Torp-Pedersen C Hofsten D Klovgaard L Early versus standard care invasive examination and treatment of patients with non-ST-segment elevation acute coronary syndrome. *Circulation.* (2018) 138:2741–50. 10.1161/CIR.0000000000000640 30565996

[3] Collet J Thiele H Barbato E Barthelemy O Bauersachs J Bhatt D 2020 ESC guidelines for the management of acute coronary syndromes in patients presenting without persistent ST-segment elevation. *Eur Heart J.* (2021) 42:1289–367. 10.1093/eurheartj/ehaa909 32860058

[4] Roffi M Patrono C Collet J Mueller C Valgimigli M Andreotti F 2015 ESC guidelines for the management of acute coronary syndromes in patients presenting without persistent ST-segment elevation: task force for the management of acute coronary syndromes in patients presenting without persistent ST-segment elevation of the european society of cardiology (ESC). *Eur Heart J.* (2016) 37:267–315. 10.1093/eurheartj/ehv320 26320110

[5] Butt J Kofoed K Kelbæk H Hansen P Torp-Pedersen C Høfsten D Importance of risk assessment in timing of invasive coronary evaluation and treatment of patients with non-ST-segment-elevation acute coronary syndrome: insights from the VERDICT trial. *J Am Heart Assoc.* (2021) 10:e022333. 10.1161/JAHA.121.022333 34585591 PMC8649124

[6] Dahlslett T Karlsen S Grenne B Eek C Sjøli B Skulstad H Early assessment of strain echocardiography can accurately exclude significant coronary artery stenosis in suspected non-ST-segment elevation acute coronary syndrome. *J Am Soc Echocardiogr.* (2014) 27:512–9. 10.1016/j.echo.2014.01.019 24612899

[7] Balfour P Jr. Gonzalez J Kramer C. Non-invasive assessment of low- and intermediate-risk patients with chest pain. *Trends Cardiovasc Med.* (2017) 27:182–9. 10.1016/j.tcm.2016.08.006 27717538 PMC5346457

[8] Siontis G Mavridis D Greenwood J Coles B Nikolakopoulou A Jüni P Outcomes of non-invasive diagnostic modalities for the detection of coronary artery disease: network meta-analysis of diagnostic randomised controlled trials. *BMJ.* (2018) 360:k504. 10.1136/bmj.k504 29467161 PMC5820645

[9] Knuuti J Ballo H Juarez-Orozco L Saraste A Kolh P Rutjes A The performance of non-invasive tests to rule-in and rule-out significant coronary artery stenosis in patients with stable angina: a meta-analysis focused on post-test disease probability. *Eur Heart J.* (2018) 39:3322–30. 10.1093/eurheartj/ehy267 29850808

[10] Genders T Coles A Hoffmann U Patel M Mark D Lee K The external validity of prediction models for the diagnosis of obstructive coronary artery disease in patients with stable chest pain: insights from the PROMISE trial. *JACC Cardiovasc Imag.* (2018) 11:437–46. 10.1016/j.jcmg.2017.02.020 28624401 PMC6815347

[11] Jang J Bhapkar M Coles A Vemulapalli S Fordyce C Lee K Predictive model for high-risk coronary artery disease. *Circ Cardiovasc Imag.* (2019) 12:e007940. 10.1161/CIRCIMAGING.118.007940 30712364 PMC6368397

[12] Zhou J Chen Y Zhang Y Wang H Tan Y Liu Y Epicardial fat volume improves the prediction of obstructive coronary artery disease above traditional risk factors and coronary calcium score. *Circ Cardiovasc Imag.* (2019) 12:e008002. 10.1161/CIRCIMAGING.118.008002 30642215

[13] Zhong P Qin J Li Z Jiang L Peng Q Huang M Development and validation of retinal vasculature nomogram in suspected angina due to coronary artery disease. *J Atheros Throm.* (2021) 29:579–96. 10.5551/jat.62059 33746138 PMC9135645

[14] Feger S Ibes P Napp A Lembcke A Laule M Dreger H Clinical pre-test probability for obstructive coronary artery disease: insights from the European DISCHARGE pilot study. *Eur Radiol.* (2021) 31:1471–81. 10.1007/s00330-020-07175-z 32902743 PMC7880945

[15] Terkelsen C Lassen J Nørgaard B Gerdes J Jensen T Gøtzsche L Mortality rates in patients with ST-elevation vs. non-ST-elevation acute myocardial infarction: observations from an unselected cohort. *Eur Heart J.* (2005) 26:18–26. 10.1093/eurheartj/ehi002 15615795

[16] Singh A Voss W Lentz R Thomas J Akhter N. The diagnostic and prognostic value of echocardiographic strain. *JAMA Cardiol.* (2019) 4:580–8. 10.1001/jamacardio.2019.1152 31042262

[17] Pryor D Shaw L Harrell F Lee K Hlatky M Mark D Estimating the likelihood of severe coronary artery disease. *Am J Med.* (1991) 90:553–62. 10.1016/S0002-9343(05)80005-1 2029012

[18] Pryor D Shaw L McCants C Lee K Mark D Harrell F Value of the history and physical in identifying patients at increased risk for coronary artery disease. *Ann Intern Med.* (1993) 118:81–90. 10.7326/0003-4819-118-2-199301150-00001 8416322

[19] Hubbard B Gibbons R Lapeyre A Zinsmeister A Clements I. Identification of severe coronary artery disease using simple clinical parameters. *Arch Intern Med.* (1992) 152:309–12. 10.1001/archinte.152.2.309 1739359

[20] Genders T Steyerberg E Alkadhi H Leschka S Desbiolles L Nieman K A clinical prediction rule for the diagnosis of coronary artery disease: validation, updating, and extension. *Eur Heart J.* (2011) 32:1316–30. 10.1093/eurheartj/ehr014 21367834

[21] Hartaigh BÓ Gransar H Callister T Shaw LJ Schulman-Marcus J Stuijfzand WJ Development and validation of a simple-to-use nomogram for predicting 5-, 10-, and 15-year survival in asymptomatic adults undergoing coronary artery calcium scoring. *JACC Cardiovasc Imag.* (2018) 11:450–8. 10.1016/j.jcmg.2017.03.018 28624402 PMC5723248

[22] Wang X Cui N Xn L Ming L. Identification of a blood-based 12-gene signature that predicts the severity of coronary artery stenosis: an integrative approach based on gene network construction, support vector machine algorithm, and multi-cohort validation. *Atherosclerosis.* (2019) 291:34–43. 10.1016/j.atherosclerosis.2019.10.001 31689620

[23] Chaudhary R Kinderytë M Chaudhary R Sukhi A Bliden K Tantry U HDL-C is a marker of coronary artery disease severity and inflammation in patients on statin therapy. *Cardiovasc Revasc Med.* (2019) 20:1001–6. 10.1016/j.carrev.2018.12.019 30626544

[24] Zheng Y Wu T Gao Y Guo Q Ma Y Zhang J A novel ABC score predicts mortality in non-ST-segment elevation acute coronary syndrome patients who underwent percutaneous coronary intervention. *Thromb Haemost.* (2021) 121:297–308. 10.1055/s-0040-1718411 33129207

[25] Radwan H Selem A Ghazal K. Value of N-terminal pro brain natriuretic peptide in predicting prognosis and severity of coronary artery disease in acute coronary syndrome. *J Saudi Heart Assoc.* (2014) 26:192–8. 10.1016/j.jsha.2014.04.004 25278720 PMC4179900

[26] Potì F Simoni M Nofer J. Atheroprotective role of high-density lipoprotein (HDL)-associated sphingosine-1-phosphate (S1P). *Cardiovasc Res.* (2014) 103:395–404. 10.1093/cvr/cvu136 24891400

[27] Wong N Nicholls S Tan J Bursill C. The role of high-density lipoproteins in diabetes and its vascular complications. *Int J Mol Sci.* (2018) 19:6. 10.3390/ijms19061680 29874886 PMC6032203

[28] Kozinski M Krintus M Kubica J Sypniewska G. High-sensitivity cardiac troponin assays: from improved analytical performance to enhanced risk stratification. *Crit Rev Clin Lab Sci.* (2017) 54:143–72. 10.1080/10408363.2017.1285268 28457177

[29] Mair J Lindahl B Hammarsten O Muller C Giannitsis E Huber K How is cardiac troponin released from injured myocardium? *Eur Heart J Acute Cardiovasc Care.* (2018) 7:553–60. 10.1177/2048872617748553 29278915

[30] Januzzi J Suchindran S Coles A Ferencik M Patel M Hoffmann U High-sensitivity troponin I and coronary computed tomography in symptomatic outpatients with suspected CAD: insights from the PROMISE trial. *JACC Cardiovasc Imag.* (2019) 12:1047–55. 10.1016/j.jcmg.2018.01.021 29550314 PMC6129434

[31] Ohtani T Ueda Y Shimizu M Mizote I Hirayama A Hori M Association between cardiac troponin T elevation and angioscopic morphology of culprit lesion in patients with non-ST-segment elevation acute coronary syndrome. *Am Heart J.* (2005) 150:227–33. 10.1016/j.ahj.2004.09.051 16086923

[32] Jensen M Pereira M Araujo C Malmivaara A Ferrieres J Degano I Heart rate at admission is a predictor of in-hospital mortality in patients with acute coronary syndromes: results from 58 European hospitals: the European hospital benchmarking by outcomes in acute coronary syndrome processes study. *Eur Heart J Acute Cardiovasc Care.* (2018) 7:149–57. 10.1177/2048872616672077 27694532

[33] Gao F Huo J She J Bai L He H Lyu J Different associations between left atrial size and 2.5-year clinical outcomes in patients with anterior versus non-anterior wall ST-elevation myocardial infarction. *J Int Med Res.* (2020) 48:300060520912073. 10.1177/0300060520912073 32252575 PMC7140218

[34] Lønborg J Engstrøm T Møller J Ahtarovski K Kelbæk H Holmvang L Left atrial volume and function in patients following ST elevation myocardial infarction and the association with clinical outcome: a cardiovascular magnetic resonance study. *Eur Heart J Cardiovasc Imag.* (2013) 14:118–27. 10.1093/ehjci/jes118 22696494

