# Supplementary material for: Development and validation of a nomogram for predicting significant coronary artery stenosis in suspected non-ST-segment elevation acute coronary artery syndrome with low-to-intermediate risk stratification
Source: Front Cardiovasc Med. 2022 Dec 19;9:1013563. doi: 10.3389/fcvm.2022.1013563 (PMC9807079; doi:10.3389/fcvm.2022.1013563)
Supplement: Supplementary file 1 [file Data_Sheet_1.docx]

Supplementary Materials

***Table of contents***

**Supplementary Figure 1.** Least Absolute Shrinkage and Selection Operator (LASSO) Analysis

**Supplementary Table 1.** Baseline comparison of patients with significant coronary artery stenosis and those without significant coronary artery stenosis

**Supplementary Table 2.** Multivariate Logistic Regression Analysis and Constructed Multivariable Predictors’ Respective Weights in the SCAS Nomogram from the Derivation Set

**Supplementary Table 3.** SCAS-nomogram comparison to UDFM and DCS model

**Criteria of low-to-intermediate Risk**

**Description of SCAS-nomogram use**

**References**

**Supplementary Figure 1.** Least Absolute Shrinkage and Selection Operator (LASSO) Analysis


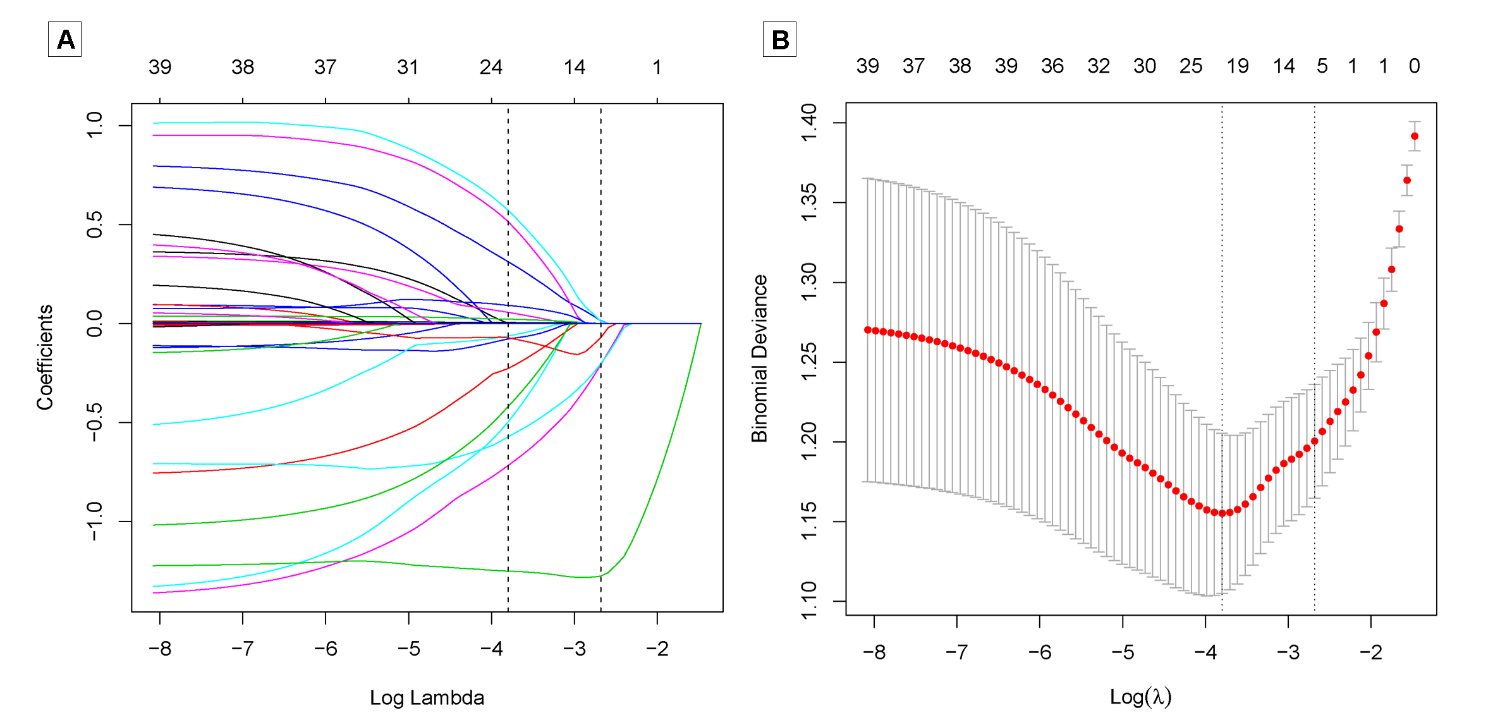


**Supplementary Table 1.** Baseline comparison of patients with significant coronary artery stenosis and those without significant coronary artery stenosis

| Characteristics | Significant coronary stenosis  Group (n=226) | Non significant coronary stenosis Group (n=243) | *t*/*χ*2/*Z* value | *P* Value |
| --- | --- | --- | --- | --- |
| Basic condition and Medical history |  |  |  |  |
| Male | 177 (78.3%) | 154 (63.4%) | 12.593 | <.001 |
| Age, mean (SD), y | 61.62 (9.78) | 58.91 (11.35) | -2.761 | .006 |
| Heart rate, mean (SD), b.p.m. | 77.62 (11.71) | 79.32 (12.14) | 1.543 | .124 |
| Systolic blood pressure, mean (SD), mm Hg | 135.68 (19.55) | 135.28 (18.26) | -0.232 | .816 |
| Diastolic blood pressure, mean (SD), mm Hg | 76.84 (12.24) | 78.45 (12.72) | 1.397 | .163 |
| Smoker | 105 (46.5%) | 63 (25.9%) | 21.477 | <.001 |
| Hypertension | 138 (61.1%) | 120 (49.4%) | 6.454 | .012 |
| Diabetes | 66 (29.2%) | 35 (14.4%) | 15.179 | <.001 |
| Previous stroke | 22 (9.7%) | 12 (4.9%) | 4.006 | .051 |
| ECG |  |  |  |  |
| Normal or Inconclusive | 75 (33.2%) | 129 (53.1%) | 37.282 | <.001 |
| TWI or AR | 67 (29.6%) | 81 (33.3%) |  |  |
| STD | 84 (37.2%) | 33 (13.6%) |  |  |
| Lab results |  |  |  |  |
| cTnT (ug/L) |  |  |  |  |
| <0.014 | 97 (42.9%) | 211 (86.8%) | 105.551 | <.001 |
| ≥0.014, <0.028 | 28 (12.4%) | 10 (4.1%) |  |  |
| ≥0.028 | 101 (44.7%) | 22 (9.1%) |  |  |
| NT-proBNP (ng/dL) |  |  |  |  |
| <300 | 157 (69.5%) | 220 (90.5%) | 34.895 | <.001 |
| ≥300, <600 | 25 (11.1%) | 12 (4.9%) |  |  |
| ≥600, <1200 | 21 (9.3%) | 7 (2.9%) |  |  |
| ≥1200 | 23 (10.2%) | 4 (1.6%) |  |  |
| Creatine Kinase, median (IQR), IU/L | 104.95 (69.60, 170.70) | 94.50 (65.50, 146.80) | -1.569 | .117 |
| Creatine Kinase MB isoform, median (IQR), IU/L | 14.55 (11.07, 20.03) | 12.60 (10.50, 16.10) | -3.630 | <.001 |
| HbA1c, mean (SD), % | 6.36 (1.32) | 6.01 (1.11) | -3.043 | .002 |
| Glucose, mean (SD), mmol/L | 7.06 (3.19) | 6.76 (3.10) | -1.047 | .296 |
| Total cholesterol, mean (SD), mmol/L | 4.77 (1.30) | 4.93 (1.13) | 1.486 | .138 |
| Triglycerides, mean (SD), mmol/L | 2.16 (1.91) | 1.95 (1.56) | -1.296 | .196 |
| HDL cholesterol, mean (SD), mmol/L | 1.09 (0.29) | 1.24 (0.35) | 4.691 | <.001 |
| LDL cholesterol, mean (SD), mmol/L | 2.94 (1.13) | 2.93 (0.94) | -0.043 | .966 |
| Homocysteine, mean (SD), μmmol/L | 13.26 (5.74) | 11.69 (4.32) | -3.360 | .001 |
| Serum uric acid, mean (SD), μmmol/L | 389.40 (103.50) | 374.07 (107.83) | -1.568 | 0.118 |
| ALAT, mean (SD), IU/L | 23.21 (12.67) | 24.75 (16.65 | 1.045 | .297 |
| ASAT, mean (SD), IU/L | 24.75 (22.61) | 24.75 (27.97) | 0.000 | 1.000 |
| eGFR, mean (SD), mL/min | 83.88 (19.70) | 87.11 (16.90) | 1.599 | .111 |
| Creatinine, mean (SD), μmmol/L | 84.16 (20.38) | 76.77 (20.15) | -3.947 | .000 |
| Medication |  |  |  |  |
| Nitrates | 61 (27.0%) | 44 (18.1%) | 5.319 | .021 |
| Platelet inhibitors | 221 (97.8%) | 200 (82.3%) | 30.555 | <.001 |
| Statins | 194 (85.8%) | 180 (74.1%) | 10.037 | .002 |
| β-Blockers | 167 (73.9%) | 114 (46.9%) | 35.491 | <.001 |
| Anticoagulant | 120 (53.1%) | 13 (5.3%) | 131.401 | <.001 |
| Calcium channel blockers | 83 (36.7%) | 76 (31.3%) | 1.552 | .242 |
| ACEI or ARB | 99 (43.8%) | 72 (29.6%) | 10.157 | .002 |
| Echocardiography |  |  |  |  |
| E/A, mean (SD) | 1.00 (0.52) | 1.04 (0.38) | 0.995 | .320 |
| LVEF, mean (SD), % | 57.34 (6.62) | 59.31 (4.53) | 3.771 | .000 |
| IVST, mean (SD), mm | 9.83 (1.50) | 9.67 (1.39) | -1.220 | .223 |
| LVPWT, mean (SD), mm | 9.75 (1.44) | 9.55 (1.37) | -1.502 | .134 |
| LVId, mean (SD), mm | 43.98 (5.73) | 43.80 (4.54) | -0.393 | .694 |
| RVId, mean (SD), mm | 20.33 (3.29) | 20.13 (3.01) | -0.669 | .504 |
| LAd, mean (SD), mm | 33.57 (5.23) | 32.40 (4.79) | -2.408 | .016 |
| RAd, mean (SD), mm | 30.48 (4.24) | 29.62 (4.00) | -2.270 | .024 |
| WMSI, mean (SD) | 1.09 (0.17) | 1.03 (0.11) | -4.404 | <.001 |
| GRACE Score, mean (SD) | 107.96 (20.00) | 96.95 (20.86) | -5.828 | <.001 |
| Abbreviations: TWI, T wave inversion; AR, arrhythmia; STD, ST-segment depression; cTnT, cardiac troponin T; eGFR, estimated glomerular filtration rate; ALAT, alanine aminotransferase; ASAT, aspartate aminotransferase; ACEI, angiotensin-converting enzyme inhibitors; ARB, angiotensin receptor blockers; E/A, early diastolic peak velocity over late diastolic peak velocity of mitral orifice; IVST, interventricular septum thickness; LVPWT, Left ventricular posterior wall thickness; LVId, Left ventricular diastolic dimension; LAd, Left atrial diameter; RAd, Right atrial diameter; WMSI, Left ventricular wall motion score index; GRACE, the Global Registry of Acute Coronary Event. | | | | |

**Supplementary Table 2.** Multivariate Logistic Regression Analysis and Constructed Multivariable Predictors’ Respective Weights in the SCAS Nomogram from the Derivation Set

|  | Estimate | Std.Error | Odds Ratio | 95%, CI. low | 95%, CI. upp | *P* value |
| --- | --- | --- | --- | --- | --- | --- |
| **Smoker** | 0.606 | 0.282 | 1.833 | 1.054 | 3.187 | .032 |
| **Diabetes** | 1.409 | 0.371 | 4.090 | 1.976 | 8.466 | <.001 |
| **Heart rate (b.p.m)** |  |  |  |  |  |  |
| <70 | Ref. |  |  |  |  |  |
| ≥70, <90 | -0.609 | 0.344 | 0.544 | 0.277 | 1.067 | .077 |
| ≥90 | -1.259 | 0.492 | 0.284 | 0.108 | 0.744 | .010 |
| **cTnT (ug/L)** |  |  |  |  |  |  |
| <0.014 | Ref. |  |  |  |  |  |
| ≥0.014, <0.028 | 1.507 | 0.462 | 4.511 | 1.824 | 11.158 | .001 |
| ≥0.028 | 1.958 | 0.364 | 7.084 | 3.470 | 14.461 | <.001 |
| **NT-proBNP (ng/dL)** |  |  |  |  |  |  |
| <300 | Ref. |  |  |  |  |  |
| ≥300, <600 | 0.705 | 0.601 | 2.023 | 0.623 | 6.577 | .241 |
| ≥600, <1200 | 1.218 | 0.653 | 3.382 | 0.941 | 12.158 | .062 |
| ≥1200 | 1.597 | 0.855 | 4.939 | 0.924 | 16.411 | .062 |
| **HDL-C (mmol/L)** |  |  |  |  |  |  |
| <0.94 | Ref. |  |  |  |  |  |
| ≥0.94, <1.14 | -0.045 | 0.407 | 0.953 | 0.431 | 2.122 | .913 |
| ≥1.14, <1.33 | -1.176 | 0.408 | 0.309 | 0.139 | 0.686 | .004 |
| ≥1.33 | -1.301 | 0.401 | 0.272 | 0.124 | 0.597 | .001 |
| **LAd (mm)** |  |  |  |  |  |  |
| <39 | Ref. |  |  |  |  |  |
| ≥39 | 1.056 | 0.479 | 2.873 | 1.124 | 7.344 | .027 |
|  | | | | | | |

|  | **AUC (95%*CI*)** | **NRI** | ***P*-value(compare) or *P*-value** |
| --- | --- | --- | --- |
| **Development Set** |  |  |  |
| **UDFM and SCAS-Nomogram** |  |  |  |
|  |  |  | <.001 |
| UDFM | 0.60 (0.53, 0.65) |  |  |
| SCAS-Nomogram | 0.83 (0.78, 0.87) |  |  |
|  |  | 32.3% | <.001 |
| **DCS and SCAS-Nomogram** |  |  |  |
|  |  |  | <.001 |
| DCS | 0.55 (0.48, 0.61) |  |  |
| SCAS-Nomogram | 0.83 (0.78, 0.87) |  |  |
|  |  | 38.8% | <.001 |
| **Validation Set** |  |  |  |
| **UDFM and SCAS-Nomogram** |  |  |  |
|  |  |  | .051 |
| UDFM | 0.62 (0.53, 0.72) |  |  |
| SCAS-Nomogram | 0.79 (0.71, 0.86) |  |  |
|  |  | 23.4% | .031 |
| **DCS and SCAS-Nomogram** |  |  |  |
|  |  |  | <.001 |
| DCS | 0.51 (0.41, 0.60) |  |  |
| SCAS-Nomogram | 0.79 (0.71, 0.86) |  |  |
|  |  | 41.1% | <.001 |
| Abbreviations: UDFM, updated Diamond-Forrester method; DCS, duke clinical score; AUC, area under the curve; NRI, net reclassification improvement. | | | |

**Supplementary Table 3.** SCAS-nomogram comparison to UDFM and DCS model

**We calculate the Updated Diamond–Forrester score and Duke score according to the following formula(1, 2)：**

**UDFM score = 1 / 〔1 + e ^－ (－ 4. 37 + 0. 04 × age + α × gender + β × type of chest pain)^ 〕**，α = 1. 34 (male) or 0 (female) ，β = 1. 91 (typical chest pain) or 0. 64 (atypical chest pain) or 0 (non-specific chest pain).

**DCS score = 1 / (1 + e^a^) 〔^a = － (－ 7. 376 + 0. 112 6 × age － α × gender － 0. 030 1 × age × gender + β × type of chest pain + 2. 596 × smoker + 0. 694 × diabetes + 1. 845 × hyperlipidemia + 1. 093 × old myocardial infarction + 1. 213 × Q wave in ECG + 0. 637 × ST-T change in ECG+ 0. 741 × old myocardial infarction × Q wave in ECG － 0. 040 4 × age × smoker － 0. 025 1 × age × hyperlipidemia + 0. 550 × gender × smoker)^ 〕,** α and β were the same as UDFM score, age >70 years old were calculated as 70 years old (the upper limit of age for DCS model). Smoker, diabetes, hyperlipidemia, old myocardial infarction, Q wave, ST-T change in ECG are all qualitative indicators (yes =1 while no = 0).

The characteristics of chest pain can be divided into: typical chest pain mainly has the following three characteristics: (1) induced by fatigue, physical exercise or emotional excitement; (2) located in the posterior sternum or anterior heart area; (3) It can be relieved within a few minutes by rest or by taking nitrates. If two of the above three characteristics are satisfied, it is defined as atypical chest pain, if only one or none of them are satisfied, it is defined as non-specific chest pain.

**Criteria of low-to-intermediate Risk**

Low-to-intermediate risk was defined as the absence of any high or very high-risk situation. Very high-risk patients (with at least one of the following criteria): 1) hemodynamic instability or cardiogenic shock; 2) recurrent or refractory chest pain despite medical treatment; 3) life-threatening arrhythmias; 4) mechanical complications of myocardial infarction; 5) heart failure clearly related to NSTE-ACS; 6) presence of ST-segment depression > 1mm in ≥6 leads additional to ST-segment elevation in aVR and/or V1. High-risk patients (with any of the following criteria): 1) GRACE risk score >140; 2) diagnosis of NSTEMI; 3) dynamic or presumably new contiguous ST/T-segment changes suggesting ongoing ischemia; 4) transient ST-segment elevation(3).


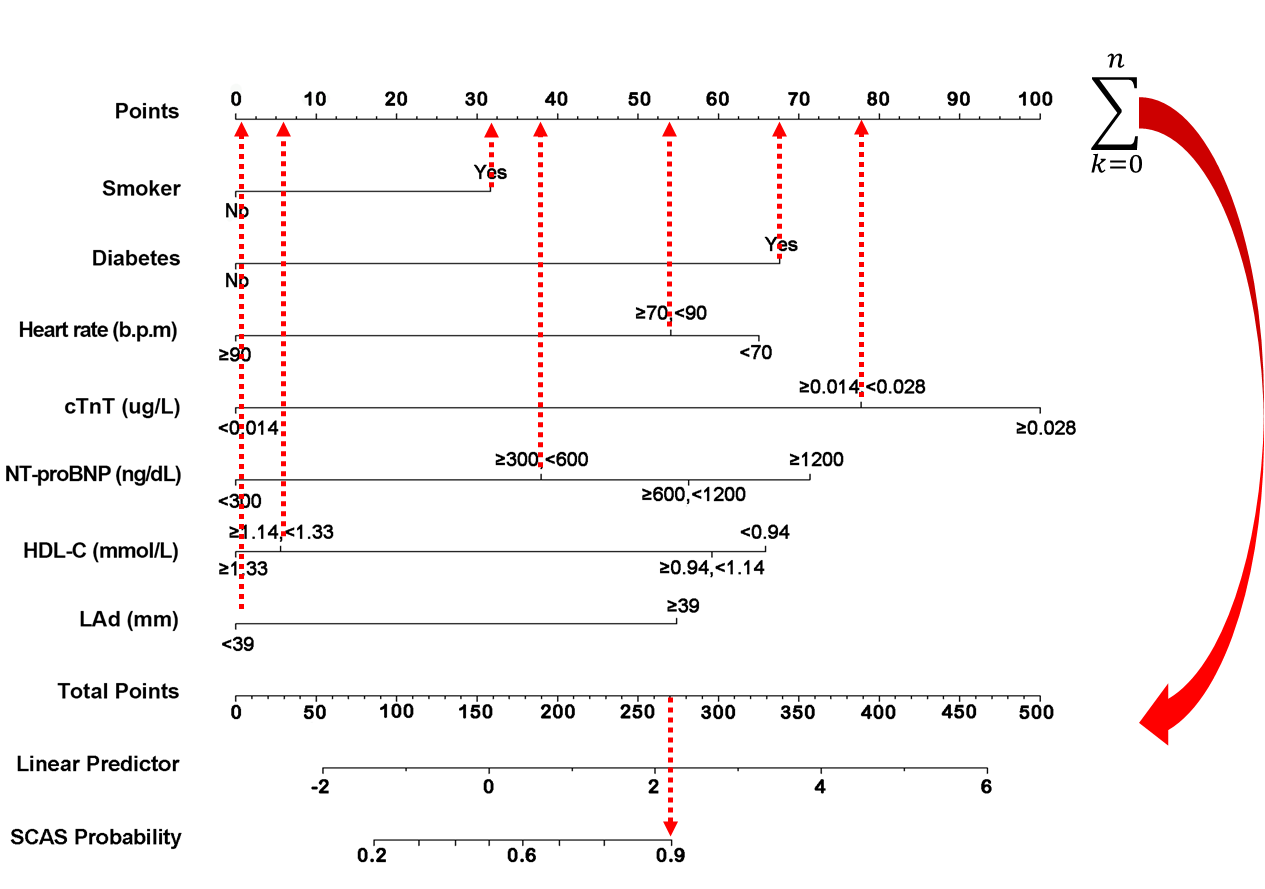
**Description of SCAS-nomogram use**

As an example, a patient with atypical chest pain and is not considered high-risk. We don't know if further coronary examination is necessary. But he/who is a smoker, diabetic with a admission heart rate at 75 b.p.m, and the basic lab results and echo shows that: cTnT 0.020 ug/l, NT-proBNP 538 ng/dl, HDL-C 1.25 mmol/L, Left atrial diameter (at left ventricular end-diastolic), will have a total score of 275 points, which corresponds to a probability of significant coronary artery stenosis of 90%. Invasive coronary angiography is recommended.

**References**

1. Genders TSS, Steyerberg EW, Alkadhi H, Leschka S, Desbiolles L, Nieman K, et al. A clinical prediction rule for the diagnosis of coronary artery disease: validation, updating, and extension. European heart journal. 2011;32(11):1316-30.

2. Pryor DB, Shaw L, McCants CB, Lee KL, Mark DB, Harrell FE, et al. Value of the history and physical in identifying patients at increased risk for coronary artery disease. Ann Intern Med. 1993;118(2):81-90.

3. Collet JP, Thiele H, Barbato E, Barthelemy O, Bauersachs J, Bhatt DL, et al. 2020 ESC Guidelines for the management of acute coronary syndromes in patients presenting without persistent ST-segment elevation. European heart journal. 2021;42(14):1289-367.
